# Improved outcome of patients with diabetes mellitus with good glycemic control in the cardiac intensive care unit: a retrospective study

**DOI:** 10.1186/s12933-019-0810-8

**Published:** 2019-01-11

**Authors:** Kassem Sharif, Suheil Ghadir, Daniela Jakubowicz, Howard Amital, Nicola Luigi Bragazzi, Abdulla Watad, Julio Wainstein, Yosefa Bar-Dayan

**Affiliations:** 1 Department of Medicine ‘B’, Sheba Medical Center, Tel-Hashomer, Israel; 2 Diabetes Unit, Wolfson Medical Center, Holon, Israel; 3 Sackler Faculty of Medicine, Tel Aviv University, Tel Aviv, Israel; 4 Postgraduate School of Public Health, Department of Health Sciences (DISSAL), University of Genoa, Genoa, Italy

**Keywords:** Diabetes mellitus, Glycemic control, Hypoglycemia, Mortality, Length of admission

## Abstract

**Background:**

Diabetes mellitus (DM) is a prevalent metabolic disease characterized by chronic hyperglycemia. A primary burden of DM is related to its long-term complications, which have been shown to impact the course of hospitalization and to influence patients’ outcome.

**Aim:**

To assess the role of in-hospital glucose control on length of stay, 30-days and 1-year mortality.

**Methods:**

This is a retrospective study that included patients admitted to the cardiac intensive care unit (CICU) of the Edith Wolfson Medical Centre between 01 January, 2010 and 31 December 2013. Blood glucose was measured by glucometer and fed into an interactive database. Glucose status was referred to as controlled when more than 50% of a given patients glucose values were between 71 and 200 mg/dL. Chisquared tests were used to assess the distribution of categorical variables, while the ttest was applied for continuous variables. A multivariate logistic regression model was used to analyze the association between glucose control and mortality. Cox regression was conducted to assess survival and 1-year mortality.

**Results:**

2466 patients were admitted to the CICU over the study period, of which 370 had concomitant diabetes mellitus. Controlled glucose status was associated with shorter length of hospital stay (1.6 ± 1.7 versus 2.6 ± 3.0, p < 0.001), reduced 30-day mortality (0.7% versus 4.6%, p < 0.001), and improved 1-year mortality (2.2% versus 7.5%, p < 0.001). Moreover, attainment of glucose control was independently associated with a significant decrease in 1-year mortality (OR = 0.371, 95% CI 0.140–0.988, p = 0.047).

**Conclusion:**

In-hospital control of glucose parameters is associated with shorter length of hospital stay, and lowered 30-day and 1-year mortality. An effort to maintain glucose levels within reference ranges is warranted in critically ill patients to reduce mortality.

## Background

Diabetes mellitus (DM) is a prevalent metabolic disease characterized by chronic hyperglycemia. The global prevalence of DM in adults is close to 8.3% in the middle age group (40–59 years) with a higher incidence in males [1]. During the past 20 years, the global prevalence of DM has doubled, and epidemiological results indicate an unsustainable increase in global expenditure related to diabetes and its complications [2].

Approximately one-quarter of hospitalized patients have diabetes. Therefore glycemic control during hospitalization has become a principal target in care management. Large fluctuations in blood sugar levels with extreme bouts of hyperglycemia and hypoglycemia are common in hospitalized patients. These fluctuations have been attributed to physical stress of illness, drugs administered, surgical procedures, changes in dietary intake, and changes in the patient’s diabetic regimen [3].

Glycemic control of patients in the intensive care units (ICUs) is of similar importance. However, the definition of optimal glycemic control in ICU remains an active avenue for research and considerable controversy exist on the optimal glycemic control among critically ill patients [4]. In the wake of tight glycemic control in the intensive care units, minimizing hypoglycemia remains a challenge as an increased mortality with higher hypoglycemic episodes in patients under intensive glucose control in ICU was observed [5].

In light of these data, we sought to investigate the link between glycemic control and both the length of hospital stay and mortality rate among patients admitted to the cardiac intensive care unit (CICU).

## Materials and patients

### Study design and population

Our study is retrospective, and includes patients admitted to the Cardiac Intensive Care Unit (CICU) at the Edith Wolfson Medical Centre between 01 January, 2010 and 31 December 2013. Edith Wolfson Medical Centre is a 650-bed governmental hospital that serves close to 500,000 residents in Israel. Patients were defined as diabetic on the basis of glycosylated hemoglobin levels, which is measured as part of a standard follow-up in primary health care. Noteworthy, in Israel participation in medical insurance is compulsory, which is provided by four large health maintenance organizations. All admitted diabetic patients were enrolled into the study. In other cases where a diagnosis has not yet been established, one is declared based on glucose value on admission. During ICU hospitalization, subjects with diabetes were treated in accordance with endocrinology consultation and recommendations. ICU patients are treated with basal bolus therapy infusion while withholding any oral hypoglycemic agents. Insulin administration included scheduled basal insulin, prandial doses, and sliding scale insulin if needed. Other data including patient demographics, cardiovascular risk factors, comorbidities, clinical and laboratory data were collected from computerized databases for all patients included in our study. The Charlson Comorbidity Index (CCI) was calculated based on comorbidities present in individual patients. Upon discharge from the ICU, the data regarding patient outcome was retrieved from ward admission and the integrated computerized virtual medical records.

### Glucose measurement and glycemic control

In 2008, Edith Wolfson Medical Centre launched a program for the treatment of hospitalized patients with diabetes by the introduction of an institutional blood glucose monitoring system (IGMS). This IGMS consists of a point-of-care automated glucometer and an interactive database. Glucose levels are measured using the automated glucometer (Accu-Chek Inform, Roche Diagnostics, Indianapolis, IN). Thereafter, data are transmitted to the central database allowing for data access, monitoring, and analysis [6].

Blood glucose was measured four times daily by continuously trained nurses. The four measurements included: fasting blood sugar, and glucose levels before the three meals (08:00, 13:00, 19:00 daily). Furthermore, measurement of hemoglobin A1c was included for all patients admitted to the study. Pre-prandial glucose levels were measured to guide pre-prandial short acting insulin injection. Fasting blood sugar readings were taken upon waking to guide long acting glucose administration. The Charlson Comorbidity Index was implemented to control for the patients respective general health status and severity of other comorbidities.

Due to the lack of clear guidelines for target glycemic control in patients admitted in intensive care setting, glucose indices were referred to as controlled when more than half of glucose values were between 71 and 200 mg/dL. Hypoglycemia was defined as glucose levels less than than 70 mg/dL. The upper limit of glycemic control was chosen based on the existing evidence from the literature on the worst patient outcomes, where glucose levels were above 200 mg/dL [7, 8]. Therefore patients were considered hyperglycemic at admission if their blood glucose metric exceeded 200 mg/dL.

### Data analysis

Statistical data analysis was carried out using SPSS version 25.0 statistical analysis software (SPSS Inc., Chicago, IL). Continuous variables were computed as mean ± standard deviation values, while categorical variables were recorded as percentages, where appropriate. The Kolmogorov–Smirnov test was employed to assess equality of continuous variables. Alpha was set at the *p*-value critical cutoff of 0.05. A Chi squared test was used to assess the distribution of categorical variables, while the *t*-test was applied for continuous variables. Correlations between continuous variables were done using either a Pearson or Spearman correlation. The association between attainment of glucose control and 30-day mortality was assessed by multivariate logistic regression. Independent variables included age, sex, and pulmonary edema at presentation, chronic renal failure, Charlson Comorbidity Index, and glucose control index. These factors were chosen due to their potential confounding effects on the desired outcome [9, 10]. Cox proportional hazard regression for survival time was implemented to assess for glucose control, 30-day, and 1-year mortality.

### Ethical approval

The study was approved by the ethical committee of Edith Wolfson Medical Centre, Holon, Israel.

## Results

### Results

Between 2010 and 2013, 2466 patients were admitted to the CICU of Edith Wolfson Medical Centre. Selected baseline characteristics of the recruited patients are presented in Table 1. The average age of the patients was 63.8 ± 13.7, 73.6% of whom were males. Table 2 lists the complications during admission, comorbidities, and baseline laboratory results. Of all patients admitted to the CICU, 2407 (97.6%) were either discharged to their homes (87.3%) or transferred to internal medicine wards (12.7%). The 30-day mortality rate in CICU patients was 1.9%, and the 1-year mortality rate was 3.9%.

**Table 1:** List of complications during admission, co-morbidities and risk factors of all the patients enrolled in the study

Acute diseases/complications	Value
Pulmonary edema (%)	5.5
Acute infection (%)	3.2
Acute renal failure (%)	1.7
Acute CVA (%)	0.2
Aspiration (%)	0.2
COPD exacerbation (%)	0.5
Co-morbidities and risk factors (%)	
Hypertension (%)	26.8
Hyperlipidemia (%)	24.3
Diabetes mellitus (%)	15.0
History of IHD (%)	12.2
Smoker (%)	4.8
Obesity (%)	4.3
Chronic renal failure (%)	3.2
Congestive heart failure (%)	2.6
Chronic obstructive pulmonary disease (%)	1.9
Peripheral vascular disease (%)	1.4
History of stroke (%)	1.0
Baseline laboratory information	
Albumin (g/dL)	3.94 ± 0.39
Creatinine (mg/dL)	1.05 ± 0.66
Cholesterol (mg/dL)	175 ± 46
Hemoglobin (g/L)	13.6 ± 1.7
WBC (cells/cmm^3^)	9.9 ± 4.4

**Table 2:** Demographic information, complication incidence and co-morbidities according to diabetes mellitus status

	No DM n = 2096	DM n = 370	p value
Age (years)	*63.1 ± 14.0*	*67.8 ± 11.0*	*<* *0.001*
Male sex	*74.6*	*67.6*	*0.005*
ICCU admission	97.7	97.3	0.672
Complications			
Pulmonary edema	5.4	5.7	0.854
Acute infection	3.1	3.8	0.459
Acute renal failure	*1.2*	*4.1*	*<* *0.001*
Exacerbation of COPD	0.5	0.5	0.969
Acute stroke	0.2	0.3	0.909
Aspiration	0.2	0.3	0.909
Co-morbidities			
Hypertension	*20.2*	*64.3*	*<* *0.001*
Hyperlipidemia	*18.4*	*57.8*	*<* *0.001*
Chronic ischemic heart disease	*9.2*	*29.2*	*<* *0.001*
Active smoker	4.7	5.4	0.544
Obesity	*2.7*	*13.0*	*<* *0.001*
Congestive heart failure	*2.1*	*5.1*	*0.001*
Chronic obstructive pulmonary disease	*1.4*	*4.9*	*<* *0.001*
Peripheral vascular diseases	*0.8*	*5.1*	*<* *0.001*
History of stroke	*0.7*	*2.7*	*<* *0.001*
Past smoker	*0.8*	*2.4*	*0.005*
Chronic renal failure	*2.3*	*8.4*	*<* *0.001*
Charlson Comorbidity Index	*0.70 ± 0.76*	*1.82 ± 1.65*	*<* *0.001*

Values in italics signify statistically significant results

In our cohort, 370 patients (15%) had diabetes mellitus, whereas 2096 did not have diabetes mellitus. Of the 370 patients with diabetes mellitus, 202 patients had acute coronary syndrome [129 patients with ST-elevated myocardial infarction (STEMI), 71 patients with non-STEMI, and two patients with unstable angina]. The remaining patients were admitted due to various cardiac indications including atrioventricular heart block, arrhythmia, pacemaker transplantation and coronary catheterization complications. Patients with diabetes were significantly older than patients without diabetes (67.8 ± 11.0 versus 63.1 ± 14.0, p < 0.001, respectively), and with higher female preponderance (32.2 versus 25.4, p = 0.005, respectively). Furthermore, patients with diabetes had increased co-morbidities with significantly higher Charlson Comorbidity Index (1.82 ± 1.65 versus 0.70 ± 0.76, p < 0.001, respectively) (Table 2).

Baseline laboratory comparative analysis in patients with DM as compared to patients without diabetes is depicted in Table 3. Patients with diabetes had significantly elevated glucose levels (213 ± 116 mg/dL versus 145 ± 71 mg/dL p < 0.001), increased creatinine values (1.15 ± 0.78 mg/dL versus 1.03 ± 0.63 mg/dL, p = 0.010) and higher cholesterol indices (165 ± 49 mg/dL versus 147 ± 45 mg/dL, p < 0.001).

**Table 3:** Baseline laboratory information of enrolled patients assorted by diabetes mellitus status

	No DM n = 2096 Mean ± SD	DM n = 370 Mean ± SD	p value
Albumin (g/dL)	*3.96 ± 0.37*	*3.83 ± 0.46*	*< 0.001*
Creatinine (mg/dL)	*1.03 ± 0.63*	*1.15 ± 0.78*	*0.010*
Cholesterol (mg/dL)	*147 ± 45*	*165 ± 49*	*< 0.001*
Hemoglobin (g/L)	*13.7 ± 1.7*	*13.0 ± 1.9*	*< 0.001*
WBC (cells/cmm^3^)	9.9 ± 4.5	9.9 ± 3.8	0.985
First glucose (mg/dL)	*145 ± 71*	*213 ± 116*	*< 0.001*

Values in italics signify statistically significant results

*SD* standard deviation, *WBC* white blood count.

### The influence of glucose control on prognostic measures in admitted patients

In the overall population, improper glucose control was associated with insurgence of pulmonary edema at presentation (4.4% versus 10.4%, in controlled and non controlled patients respectively, p < 0.001), acute renal failure (0.9% versus 3.9%, p = 0.001), acute infection (2.4% versus 7.5%, p < 0.001), Charlson Comorbidity Index (0.7 ± 0.8 versus 0.9 ± 0.8, p = 0.002), 30-day mortality (0.7% versus 4.6%, p < 0.001), and 1-year mortality (2.2% versus 7.5%, p < 0.001). Moreover, subjects with poor glucose control were older than individuals with controlled glucose (67.5 ± 12.7 versus 62.4 ± 13.9, p < 0.001), with higher creatinine values (1.3 ± 1.0 versus 1.0 ± 0.6, p < 0.001), a higher WBC count (11.6 ± 5.3 versus 9.6 ± 4.3, p < 0.001), lower hemoglobin (13.2 ± 1.9 versus 13.8 ± 1.6, p < 0.001), and lower serum albumin concentrations (3.8 ± 0.4 versus 4.0 ± 0.4, p < 0.001).

### The influence of glucose control on prognostic measures in admitted diabetic patients

Of the 370 diabetic patients, 343 (92.7%) patients had glucose test results entered in the electronic records and were included in the analysis. 166 (48.8%) patients had more than 50% of glucose values between 71 and 200 mg/dL and thus were defined as patients with controlled diabetes mellitus. Table 4 includes demographic data, acute illness and co-morbidities of patients according to glucose control status. Patients with controlled glucose levels were older (69.0 ± 10.8 versus 66.5 ± 11.0, p = 0.035) and with lower Charlson Comorbidity Index (1.59 ± 1.67 versus 2.05 ± 1.67, p = 0.011) as compared to patients with poor glucose control.

**Table 4:** Comparison of demographics, acute illnesses and co-morbidities according to glucose control status

	Over 50% of glucose between 71 and 200 mg/dL		
	No n = 177 Mean ± SD	Yes n = 166 Mean ± SD	p value
Age (years)	*66.5 ± 11.0*	*69.0 ± 10.8*	*0.035*
Male sex (%)	63.8	72.9	0.072
Acute illnesses during admission			
Acute infection (%)	5.6	2.4	0.130
Acute renal failure (%)	6.2	2.4	0.085
Pulmonary edema (%)	6.8	4.8	0.439
Acute stroke (%)	0.6	0	0.322
Co-morbidities and risk factors			
Hypertension (%)	*70.1*	*57.2*	*0.013*
Hyperlipidemia (%)	61.6	55.4	0.247
Obesity (%)	13.6	9.6	0.258
Chronic renal failure (%)	8.5	8.4	0.989
Smoking (%)	7.9	2.4	0.022
CHF (%)	6.8	3.6	0.189
History of stroke (%)	4.0	1.8	0.237
Endocrine (%)	3.4	3.6	0.910
Past smoker (%)	2.3	2.4	0.927
Charlson Comorbidity Index	*2.05 ± 1.67*	*1.59 ± 1.67*	*0.011*

Values in italics signify statistically significant results

*SD* standard deviation

Comparison of laboratory data according to glucose control status is presented in Table 5. When comparing patients with controlled versus uncontrolled glucose status, it is evident that the former group had a lower average glucose levels (138 ± 33 mg/dL, 123 [95% CI 104–156] versus 221 ± 68 mg/dL, 178 [95% CI 135–256], p < 0.001), lower hypoglycemic episodes (0.6% versus 4.5%, p = 0.023), lower HbA1c (6.7 ± 1.1 versus 8.1 ± 1.8, p < 0.001), fewer episodes of glucose over 400 mg/dL (0% versus 4.0%, p = 0.010), and lower creatinine levels (1.03 ± 0.52 versus 1.25 ± 0.95, p = 0.013).

**Table 5:** Comparison of laboratory data according to glucose control status in diabetic patients admitted to the CICU

	Over 50% of glucose between 71 and 200 mg/dL		
	No n = 177 Mean ± SD	Yes n = 166 Mean ± SD	p Value
First glucose (mg/dL)	*263 ± 129*	*160 ± 67*	*< 0.001*
Average glucose (mg/dL)	*221 ± 68*	*138 ± 33*	*< 0.001*
Hypoglycemia (%)	*4.5*	*0.6*	*0.023*
Glucose over 400 mg/dL (%)	*4.0*	*0*	*0.010*
^a^HbA1c (%)	*8.1 ± 1.8*	*6.7 ± 1.1*	*< 0.001*
Albumin (g/dL)	*3.7 ± 0.5*	*3.9 ± 0.4*	*< 0.001*
Creatinine (mg/dL)	*1.25 ± 0.95*	*1.03 ± 0.52*	*0.013*
Cholesterol (mg/dL)	167 ± 52	162 ± 45	0.380
Hemoglobin (g/L)	12.8 ± 1.9	13.1 ± 1.9	0.204
WBC (cells/cmm^3^)	*10.5 ± 4.2*	*9.2 ± 3.0*	*0.001*

Values in italics signify statistically significant results

^a^42.4% of the “No” group and 40.1% of the “Yes” group had documented HbA1c levels

*HbA1c* glycosylated hemoglobin

A statistically significant association was found between the attainment of glucose control and severity of diabetes mellitus: controlled glucose status was achieved in 82.0%, 54.6% and 44.0% of mild, moderate and severe diabetes, respectively (p < 0.001, χ^2^ for trend p < 0.001).

Controlled glucose status was additionally associated with shorter length of admission (1.6 ± 1.7 versus 2.6 ± 3.0, p < 0.001), decreased 30-day mortality rate (2.4% versus 8.5%, p = 0.014), and decreased 1-year mortality rate (4.8% versus 11.9%, p = 0.019).

To assess the influence of glucose control on 30-day mortality two logistic regression models were built. Independent variables included age, sex, pulmonary edema at presentation, chronic renal failure, Charlson Comorbidity Index, admission due to acute coronary syndrome, and glucose control index. Previous studies on critically ill patients suggest the association of pulmonary edema and chronic renal failure on increased mortality rate [9, 10]. In our current study, pulmonary edema at presentation was shown to be independently associated with increased 30-day mortality (OR 8.152, 95% CI 2.395–27.751, p = 0.001). Furthermore, controlled glucose measures (> 50% of glucose values between 71 and 200 mg/dL) were independently associated with decreased 30-day mortality in a borderline way (OR = 0.312, 95% CI 0.092–1.057, p = 0.061). When adjusting also for HbA1c values, only pulmonary edema (OR = 31.865, 95% CI 2.426–418.567, p = 0.008) was associated with 30-day mortality.

Regarding 1-year mortality, pulmonary edema at presentation (OR = 9.255, 95% CI 3.024–28.321, p < 0.001), Charlson Comorbidity Index (OR = 1.290, 95% CI 1.011–1.646, p = 0.041), and glucose control (OR = 0.371, 95% CI 0.140–0.988, p = 0.047) were found to be independent statistical predictors. When adjusting for HbA1c, only pulmonary edema resulted an independent predictor (OR = 53.043, 95% CI 5.294–531.465, p = 0.001).

It is noteworthy that 33.6% of subjects with pulmonary edema had poor glucose control versus 19.4% of individuals without pulmonary edema (p < 0.0001).

**Fig. 1:**
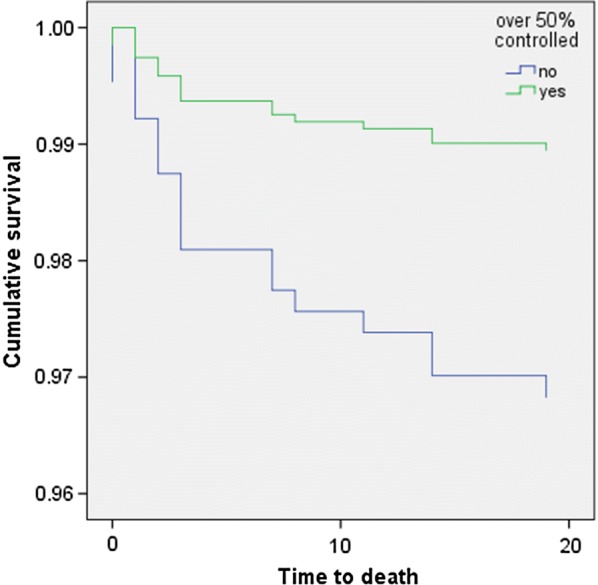
Cox 30-day survival curve according to attainment of 50% of glucose between 70 and 200 mg/dL during hospitalization

**Fig. 2:**
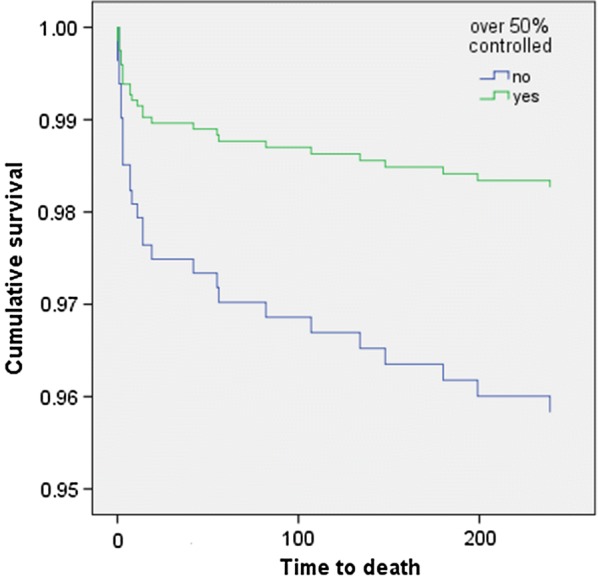
Cox 1-year survival curve according to attainment of 50% of glucose between 70 and 200 mg/dL during hospitalization

Stratifying according to diabetes mellitus status, the Cox survival curve shows that pulmonary edema at presentation (HR = 5.831, 95% CI 2.059–16.514, p = 0.001) was significantly associated with 30-day mortality, whereas glucose control (HR = 0.329, 95% CI 0.104–1.044, p = 0.059) was a statistically borderline independent predictor. When adjusting for HbA1c, only pulmonary edema (HR = 13.491, 95% CI 1.728–105.350, p = 0.013) was found to be associated (Fig. 1, Table 6). Furthermore, attainment of glucose control was independently associated with a significant decrease in 1-year mortality when controlling for age, sex, pulmonary edema at presentation, smoking, CCI, and admission due to acute coronary syndrome (HR = 0.410, 95% CI 0.174–0.967, p = 0.042) (Fig. 1). CCI was not associated with increased 1-year mortality (HR = 1.221, 95% CI 0.998–1.494, p = 0.053). Pulmonary edema at presentation was also shown to be independently associated with increased 1-year mortality (HR = 6.011, 95% CI 2.565–14.089, p < 0.001). When adjusting for HbA1c, only pulmonary edema remained associated (HR = 24.423, 95% CI 4.981–119.751, p < 0.001), as reported in Table 7 and shown in Fig. 2.

**Table 6:** Cox multivariate regression analysis investigating covariates associated with 1-year mortality

	B	SE	Wald	Sig.	HR	95% CI HR	
						Lower	Upper
30-day mortality (model without HbA1c)							
Sex	0.703	0.516	1.856	0.173	2.019	0.735	5.548
Age	0.031	0.027	1.312	0.252	1.031	0.978	1.087
Pulmonary edema	1.763	0.531	11.018	0.001	5.831	2.059	16.514
Chronic renal failure	− 0.281	0.845	0.111	0.739	0.755	0.144	3.953
Smoking	− 12.577	549.709	0.001	0.982	0.000	0.000	0.000
Charlson comorbidity index	0.083	0.135	0.373	0.541	1.086	0.833	1.416
Hypoglycemia	− 0.297	1.065	0.078	0.780	0.743	0.092	5.994
Glucose control	− 1.112	0.589	3.562	0.059	0.329	0.104	1.044
Acute coronary syndrome	− 0.371	0.502	0.545	0.460	0.690	0.258	1.847
30**-**day mortality (model with HbA1c)							
Sex	1.944	1.278	2.315	0.128	6.990	0.571	85.555
Age	0.143	0.079	3.279	0.070	1.154	0.988	1.348
Pulmonary edema	2.602	1.049	6.157	0.013	13.491	1.728	105.350
Chronic renal failure	− 13.134	365.269	0.001	0.971	0.000	0.000	0.000
Smoking	− 9.011	483.484	0.000	0.985	0.000	0.000	0.000
Charlson comorbidity index	0.197	0.303	0.423	0.516	1.218	0.672	2.207
Hypoglycemia	− 14.399	12,337.418	0.000	0.999	0.000	0.000	0.000
Glucose control	0.442	1.359	0.106	0.745	1.556	0.109	22.313
Acute coronary syndrome	− 0.413	1.247	0.110	0.740	0.661	0.057	7.624
HbA1c	0.204	0.410	0.248	0.619	1.227	0.549	2.741

**Table 7:** Cox multivariate regression analysis investigating covariates associated with 1-year mortality

Parameter	B	SE	Wald	Sig.	HR	95% CI HR	
						Lower	Upper
1-year mortality (model without HbA1c)							
Sex	0.742	0.412	3.237	0.072	2.099	0.936	4.709
Age	0.049	0.022	4.791	0.029	1.050	1.005	1.097
Pulmonary edema	1.794	0.435	17.033	0.000	6.011	2.565	14.089
Chronic renal failure	− 0.350	0.627	0.311	0.577	0.705	0.206	2.407
Smoking	− 12.258	451.126	0.001	0.978	0.000	0.000	0.000
Charlson comorbidity index	0.199	0.103	3.748	0.053	1.221	0.998	1.494
Hypoglycemia	− 0.705	1.049	0.452	0.501	0.494	0.063	3.861
Glucose control	− 0.891	0.437	4.150	0.042	0.410	0.174	0.967
Acute coronary syndrome	− 0.121	0.403	0.090	0.765	0.886	0.403	1.952
1-year mortality (model with HbA1c)							
Sex	1.280	0.750	2.910	0.088	3.595	0.826	15.640
Age	0.130	0.047	7.751	0.005	1.139	1.039	1.248
Pulmonary edema	3.196	0.811	15.518	0.000	24.423	4.981	119.751
Chronic renal failure	− 1.133	1.619	0.490	0.484	0.322	0.013	7.682
Smoking	− 12.752	1066.916	0.000	0.990	0.000	0.000	0.000
Charlson comorbidity index	0.187	0.196	0.919	0.338	1.206	0.822	1.770
Hypoglycemia	− 14.473	7187.596	0.000	0.998	0.000	0.000	0.000
Glucose control	− 0.442	0.918	0.232	0.630	0.643	0.106	3.882
Acute coronary syndrome	− 0.075	0.810	0.009	0.926	0.928	0.190	4.537
HbA1c	0.249	0.253	0.966	0.326	1.282	0.781	2.105

## Discussion

Glycemic control during hospitalization provides a nontrivial concern in clinical outcome. In our study, we opted for a wide range of glucose concentrations that are reflective of values encountered in the daily practice. In our study, good glycemic control as defined in our study was associated with shorter hospitalization duration and a lower 30-day and 1-year mortality.

In our analysis, DM patients with 50% or more of their respective glycemic indices between 71 and 200 mg/dL had shorter length of admission as compared to uncontrolled patients with DM in the CICU (1.6 ± 1.7 days versus 2.6 ± 3.0, p < 0.001). Several studies reflect the impact of hyperglycemia and patient outcomes. Gebreegziabher et al. [11] conducted a prospective cohort study addressing glycaemia and length of hospital stay (LOS) in patients admitted for acute heart failure exacerbation. A significantly longer LOS was noted in patients with DM as compared with patients without diabetes (5.0 ± 0.29 vs 3.4 ± 0.19; *p *< .001). Moreover, LOS was significantly correlated to blood glucose at admission after correction for comorbidities (r = 0.31; *p *< .001). Similarly, in the cohort study by Targher et al. [12], elevated admission blood glucose level were associated with poor survival outcome as indicated by increased in-hospital mortality amongst patients admitted with acute heart failure. Novo et al. [13], investigated the in-hospital stay for patients admitted for acute coronary syndrome in patients with or without diabetes mellitus. Type 2 diabetes was prevalent in close to 31% of the cases, and the average hospital stay was significantly longer in patients with diabetes versus patients without diabetes (*p *< 0.005). Moreover, patients with DM and acute coronary syndrome had significantly more complications as compared to non-diabetic patients (41.1% vs 17.9%, *p *= 0.0001). It is worth noting that intensive glycemic control does not significantly improve outcome as compared to conservative control. Umpierrez et al. [14], showed that intensive glycemic control to target of 100–140 mg/dL in the ICU was not associated with better perioperative course as compared to a less conservative control after CABG surgery. This findings is corroborated by Chen et al. [15], who demonstrated a U-shaped relationship between glycemic control and cardiovascular mortality, showed that both strictly controlled and poorly controlled patients had significant worse outcome in patients admitted with acute heart failure. In contrast to other studies that demonstrate admission plasma glucose level even after adjustment for HbA1c, to be a prognostic factor associated with mortality in myocardial infarction, our study showed that upon controlling for HbA1c the result decreased to non-significant levels. This suggests the role of chronic glycemic control on mortality outcome. Further research is warranted to better elucidate this finding.

Similarly, ample evidence points toward the role of hyperglycemia in increasing morbidity and mortality in various patient populations, including hemorrhagic and ischemic stroke, pneumonia, mechanically ventilated patients, and patients undergoing CABG [16–19].

In our current study, glucose control was demonstrated to be independently associated with decreased mortality (OR 0.286, 95% confidence interval 0.086–0.951, p = 0.041). Moreover, attainment of good glucose control was associated with decreased 30-day (8.5% versus 2.4%, p = 0.014) and 1-year mortality (11.9% versus 4.8%, p = 0.019). In a prospective cohort study conducted by Zadok et al. [7], the 10-year outcome of patients with DM admitted with heart failure was investigated. The cumulative probability of mortality was significantly higher amongst patients with diabetes as compared to non-diabetic patients (85% versus 78%, *p *< 0.001). Among patients with diabetes, glucose levels above 200 mg/dL were associated with increased morality (HR = 1.20, *p *= 0.032) [7]. In another national representative study, severe hyperglycemia (> 200 mg/dL) in patients with DM was associated with elevated risk for mortality among patients with diabetes admitted for acute myocardial infarction even after adjustment for patients characteristics (OR = 3.92, 95% CI [3.04–5.04]) [8]. Furthermore, in a heterogeneous group of 1826 critically ill patients, mean and maximal glucose values were significantly higher among non-survivors versus survivors (*p *< 0.001).

The salient effect of hyperglycemia on mortality and morbidity could not be underestimated. However, observational studies identified other domains of glycemic control that are associated with increased mortality, the most notably of which is hypoglycemia. In the present study, 4.5% of patients with uncontrolled diabetes had hypoglycemic episodes as compared to 0.6% of patients with diabetes and controlled glucose readings (*p *= 0.023). In a large prospective cohort of 6240 patients, Krinsley et al. [20] demonstrated that mild hypoglycemia (blood glucose < 70 mg/dL) was significantly associated with increased mortality even after controlling for severity of illness, diagnostic category, diabetic status, and mean blood glucose levels at admission or during hospitalization (OR 1.78, 95% CI 1.39–2.27, p < 0.0001). In a retrospective database study involving case control analysis, 102 patients with at least one episode of severe hypoglycemia were recruited [21]. Mortality amongst patients with severe hypoglycemia was 55.9% as compared to 39.5% of controls (p = 0.0057). In the multivariate logistic regression model, even a single episode of severe hypoglycemia was independently associated with increased mortality (OR 2.28 95% CI 1.41–3.70, p = 0.0008 [21]. Those studies reflect the detrimental effects of extreme blood glucose deviations.

Another important concept in glycemic control is glycemic variability, which corresponds to the fluctuations of blood glucose throughout the day [22]. Glycemic variability has been shown to be associated with clinical implications. Takahashi et al. demonstrated that glycemic variability was an integral component in the progression of coronary artery disease and was determined to a predictor of prognosis with acute coronary syndrome [23]. Glycemic variability is known to be a trigger for increased oxidative stress promoting inflammation and endothelial dysfunction [24]. Another new concept is stress hyperglycemia, which is defined as the relative increase of glucose due to concurrent illness as compared to background glycaemia [25]. Relative glycaemia has been shown to be independently associated with complications after acute myocardial infarction. It is still undetermined whether the control of this relative glycaemia would result in improved outcome among patients [26].

The mechanism behind the increased mortality in patients with hypoglycemia and hyperglycemia remains to be elucidated. Prolonged hypoglycemia has been shown to result in brain death due to glutamate release [27]. Cardiac arrhythmias have been proposed as the probable cause of the majority of episodes of fatal hypoglycemia [27, 28]. Accumulating evidence points towards the activation of the sympathoadrenal response, and reduction of baroreceptor sensitivity, the latter which results in increased fatal arrhythmia [27]. On the other hand, hyperglycemia has been proven to possess toxic effects by directly influencing the immune system and circulating inflammatory cytokine concentrations [29]. Finally, hyperglycemia is associated with uncontrolled diabetes mellitus which is well established to result in microvascular and macrovascular complications that are associated with increased morbidity and mortality [30].

Our study has several limitations including its retrospective design. We used point-of-care blood glucose levels in this study and could not check the duration of the hypoglycemic or hyperglycemic event, which might be significant. Finally, the gathered data relied on a single academic hospital, which could limit generalizability.

Strengths of this study include the wide glucose range which provided an opportunity to evaluate the impact of real-hospital glucose range on patients outcomes, its large population size, use of point-of-care automated glucometer, and multiple glucose measurements that allow for a detailed analysis of glucose status.

In conclusion, diabetes mellitus is a chronic prevalent metabolic disease, which has been shown to complicate patient’s hospital course and influence patient’s outcome. Controlling glucose parameters during the course of hospital admission is associated with shorter length of hospital admission, lower 30-day mortality, and lower 1-year mortality. Efforts to maintain glucose levels within comparatively wide reference ranges are warranted, especially in critically ill patients, in order to reduce morbidity and mortality.

## Abbreviations

DM: diabetes mellitus
ICU: intensive care unit
CICU: cardiac intensive care unit
IGMS: institutional blood glucose monitoring system
STEMI: ST-elevated myocardial infarction
LOS: length of hospital stay
